# Positioning Ethics When Direct Patient Care is Prioritized: Experiences from Implementing Ethics Case Reflection Rounds in Childhood Cancer Care

**DOI:** 10.1007/s10730-024-09541-6

**Published:** 2024-11-02

**Authors:** Pernilla Pergert, Bert Molewijk, Cecilia Bartholdson

**Affiliations:** 1 Childhood Cancer Research Unit, Department of Women’s & Children’s Health, Karolinska Institutet, Stockholm, Sweden; 2 Centre for Research Ethics & Bioethics (CRB), Department of Public Health and Caring Sciences, Uppsala University, Uppsala, Sweden; 3 Dep. Ethics, Law & Humanities, Amsterdam University Medical Centers, Amsterdam, the Netherlands; 4 Centre for Medical Ethics, University of Oslo, Oslo, Norway; 5 Astrid Lindgren Children´s Hospital, Karolinska University Hospital, Stockholm, Sweden

**Keywords:** Ethics case reflection rounds, Ethics support, Grounded theory, Implementation, Moral case deliberation, Qualitative

## Abstract

Caring for children with cancer involves complex ethical challenges. Ethics Case Reflection (ECR) rounds can be offered to support teams to reflect on challenges and what *should* be done in patient care. A training course, for facilitators of ECR rounds, has been offered to healthcare professionals (HCPs) in childhood cancer care by a Nordic working group on ethics. During/after the course, the trainees implemented and facilitated ECR rounds in their clinical setting. The aim was to explore the trainees’ experiences of implementing ECR rounds in childhood cancer care. HCPs, who participated as trainees in the course, participated in 3 focus group interviews (*n* = 22) and 27 individual interviews (*n* = 17). Interview data were analysed concurrently with data collection following classic grounded theory. *Positioning ethics* is the core category in this study, used to resolve the main concern of *doing ethics* in a context where direct patient care is prioritized. Being able to take time for ethics reflections, not perceived as the key priority, was considered a luxury in the clinical setting. Strategies for positioning ethics include *allying*,* promoting ethics reflection*,* scheduling ethics reflection*, and *identifying ethical dilemmas.* These strategies can be more or less successful and vary in intensity. The prioritisation of direct patient care is not surprising, but polarisation between care and ethics needs to be questioned and ethics reflection need to be integrated in standard care. Ethical competence seems to be central in doing ethics and more knowledge on the promotion of ethical competence in practice and education is needed.

## Introduction

Healthcare is increasingly complex and involves not only medical/care uncertainties and challenges but also economical, organisational, legal and ethical uncertainties and challenges. Caring for children with cancer involves dealing with ethical uncertainties and challenges on a daily basis (Weiner et al., 2022). More and more, clinical ethics support (CES) interventions are implemented in order to help healthcare professionals (HCPs) in reflecting on their ethical uncertainties and challenges (Pedersen et al., 2010; Rasoal et al., 2017; Schildmann et al., 2017). CES aims to assist stakeholders in healthcare to deal with ethical challenges and reflecting on what should be done in treatment and care to jointly foster a good quality of care. CES also aims to assist HCPs to cope with different ethical viewpoints on good care in a constructive manner (Bartholdson et al., 2016). HCPs in childhood cancer care have expressed a need for CES, including time and reflection as well as education, in order to deal with their daily ethical challenges (Bartholdson et al., 2015). There are several methods for offering CES (Pedersen et al., 2010) such as: ethics committees, education/training, ethics consultations, moral counselling, and Moral Case Deliberation (Molewijk et al., 2016; Stolper et al., 2016), also called and henceforth referred to as Ethics Case Reflection (ECR) rounds (Bartholdson et al., 2018) or in short ethics reflection. ECR rounds are similar to interprofessional care conferences, but focusing on the ethics as stakeholders reflect on an ethical challenge that they experience in a clinical case. Trained facilitators guide the group reflection using a structured reflection model (Bartholdson et al., 2014; Stolper et al., 2015).

International research, evaluating ECR rounds, indicate that they can improve team cooperation (Bartholdson et al., 2016; Grönlund et al., 2016; Hem et al., 2018; Weidema et al., 2013) and moral awareness of HCPs (de Snoo-Trimp et al., 2022). A systematic review on the impact of ECR rounds, covering 25 empirical evaluation papers reported that ECR rounds can bring about improvements in inter-professional interactions (Haan et al., 2018). Furthermore, related to the specific characteristics of ECR rounds (i.e. learning from different viewpoints in constructive and respectful dialogues and putting yourself in someone else’s shoes), evaluation studies reported a more constructive handling of disagreement in teams (Hem et al., 2018; Molewijk et al., 2023). Finally, as ECR rounds include elucidation of values and norms of patients and their families, and ethical challenges from their perspectives, it has been suggested that the understanding of the viewpoints of patients and next of kin increased (Janssens et al., 2015; Lillemoen & Pedersen, 2015).

In the Nordic Society of Paediatric Haematology and Oncology (NOPHO) an interprofessional working group on ethics was constituted in 2008. The members of the working group on ethics are nurses, physicians, and researchers in Nordic childhood cancer care. The working group is a competence group addressing ethical challenges, offering CES, and performing research on clinical ethics in childhood cancer care. The members themselves have undergone training in facilitating ECR rounds by VU Medical Centre including a 3-days introductory workshop/course and 2-days continued training (Stolper et al., 2015). A special interest group, with two paediatric nurses and two paediatric oncologists, within NOPHO working group on ethics developed a course for facilitators of ECR rounds for Nordic HCPs in childhood cancer care, in collaboration with the ethics support group from Amsterdam University Medical Centres (Amsterdam UMC) and the VU University. In 2017, the newly developed course was offered to 29 HCPs from various Nordic childhood cancer centres. This was a unique experience since the facilitators were all working in similar clinical contexts. The management of the childhood cancer care centres supported the course as part of the implementation process. During and after the course, the trainees implemented and facilitated ECR rounds in their clinical setting. Hence, the trainees became responsible not only for facilitation but also for implementation. Therefore, it is important to gain empirical knowledge about their experiences of the implementation which can inform others to plan the implementation of ECR rounds, overcome possible barriers, and enrich future courses for facilitators of ECR rounds. The aim of this study was to explore the trainees’ experiences of implementing ECR rounds in Nordic childhood cancer care.

## Methods

In this qualitative study, classic grounded theory methodology was chosen to explore how participants dealt with their main concerns while implementing ECR rounds (Glaser, 1998; Glaser & Strauss, 1967).

### Context

The course for facilitators consisted of three parts: the first part was a 3-day introduction to guiding ECR rounds, and the second part was a 2-day follow-up where participants could fine-tune their knowledge and skills. The third part included practice periods where participants were expected to practice in their clinical setting in-between and after the two parts (i.e., performing about 4–8 ECR rounds as facilitators). Participants in the first cohort of the course included: nurses, physicians, and social worker from all 6 paediatric oncology centres in Sweden (*n* = 21) as well as nurses and physicians from other Nordic countries (*n* = 8). The Swedish trainees implemented ECR rounds at the 6 paediatric oncology centres in Sweden, in collaboration with managers, using cases from their everyday clinical practice.

The first and second author was part of the team of educators, and the first author was a member of the Nordic working group on ethics. The last author was not a member of the working group on ethics or the team of educators when conducting the individual interviews.

### Participants and Data Collection

All trainees, who participated in the first cohort of the facilitator course, were invited to participate in this study. Three focus group interviews were conducted in person with trainees (*n* = 22) from Sweden, Denmark, Finland, Iceland, and Norway that were present at the beginning of the second part of the course. Each focus group interview was conducted by two researchers (teachers in the course and the last author); one acting as moderator using an interview guide encouraging interaction and discussions in the group, and the other as an observer taking fieldnotes (Bloor et al., 2001).

Furthermore, a total of 27 individual semi-structured telephone interviews were conducted by the last author with participants (*n* = 17/21) from all paediatric oncology centres in Sweden. Two trainees declined to participate because of lack of time and two had ended their positions. The individual interviews were performed three months after the first part (*n* = 14) and the second part (*n* = 13) of the course. Field noted were written and all interviews were sound-recorded and transcribed.

### Data Analysis

Data was analysed using classic grounded theory, which meant that data collection and analysis were carried out in parallel (Glaser, 1978, 1998; Glaser & Strauss, 1967). Fieldnotes and transcribed text were read through by the last author to get an overall picture of what the respondents expressed in the focus groups and individual interviews. After that the last author started open coding line-by-line, using a software for qualitative analysis (NVivo), and two preliminary core categories emerged. One core category, which is not presented in this manuscript, was “Carrying the facilitator responsibility” which refers to their behaviour during the guiding/facilitating of the ECR rounds. The other preliminary core was “Finding a place for ethics when care is prioritized” and this was about what they were doing as they were trying to implement ECR rounds in the clinic. The first author started selective coding of data for incidents, codes and categories related to this category. Codes and categories were constantly compared and were discussed among the authors. Memos were written about the categories and their properties. Furthermore, sorting of memos and theoretical coding were done manually to integrate the categories (Glaser, 1978).

## Findings

Positioning ethics is the core category in this study, used to resolve the main concern of doing ethics, that is, conducting and participating in ethics reflections. Positioning ethics is about finding the time and the place for ethics reflection, thus, putting ethics reflections on the agenda, as exemplified by this quote “I have to do this as a scheduled position… ethics work should be in the schedule.” Positioning ethics is also about giving a status to ethics reflections within the care organisation as an integrated part of standard care. As one participant said, “In the future, we want to strive for this to become like standard care.” Thus, positioning ethics is about establishing a place for ethics reflections integrated into the clinical organization.

Positioning ethics is done in a context where direct patient care is prioritized. “…[Ethics] it’s just not a priority. I mean the priority is that a nurse and a doctor can take care of the children…”. Being able to take time for ethics reflections that are not the prioritized activities (direct patient care), but still necessary for patient care, were considered a luxury. “…we always put the children first and their care… people would easily think that this [ECR rounds] would be such a luxury to do… So that’s the mind-set we are struggling with.” Thus, the higher the clinical workload (and the higher the need for ethics reflections) the less the time for ECR rounds.

Strategies for positioning ethics include *allying*,* promoting ethics reflection*,* scheduling ethics reflection*, and *identifying ethical dilemmas*. These vary in intensity and can be more or less successful.

### Allying

Allying is about forming an alliance, which involves collaborating and sharing responsibility of positioning ethics. Allying is a necessary strategy and is done with co-facilitators and key actors, including the management.

Being a team of facilitators were especially important as one participant said, “…we [facilitator trainees] help each other, and I think we complement each other.” The key actors included enthusiastic persons making sure that ECR rounds were performed. Regarding support from managers, there was a great variation; some experienced great support while others lacked support. When the manager was interested in ethics often great support was given. There was also variation in the kind of support given by managers, including support in promoting and scheduling ethics reflection. When there was a lack of support, participants handled it by themselves, allying with other key actors. Participants in our study describe that the responsibility of positioning ethics and implementing ECR rounds should be of both facilitators and managers. “And our managers…they support us, but they will never be the driving force… and that’s not really their job”.

Allying was also about trying to promote ethics to new participants and striving for a mixed group with different professionals attending ECR rounds. As one participant said, “we have managed to get a very mixed group. We have had the senior physicians, managers, social-workers, psychologists, nursing assistants, and nurses in a mixed group.” Striving for different professions to attend was another challenge. In our study, participants describe that nurses were the most involved and there was a wish for social-workers and physicians to attend more often. At some centres physicians had become more engaged because a physician was part of the facilitator-team. Some HCPs were crucial for the ECR round to take place, for example the physician involved in and responsible for the treatment of the patient. “And then we have made sure that a doctor who is involved, the doctor who is involved is familiar with the case and can contribute with medical knowledge [facts in the case].”

### Promoting Ethics Reflection

Promoting ethics reflection is about communicating the positive qualities of ECR rounds and persuading others of the benefits of ECR rounds by presenting, representing and using self-experienced ethics reflection, as explained below.

Presenting ethics reflection: The strategy of promoting ethics reflection was done in different ways by the participants. One way was to describe and talk about the benefits of ECR rounds at meetings. As one participant said, “we have sent out…an email to invite ourselves to…meetings… So maybe it attracts… more people also get to see what it [ECR rounds] means.” However, participants expressed that it was sometimes hard to reach out to everybody working at the ward and that information about the ECR rounds was not spread sufficiently. Some participants experienced that they had to nag about the importance of ECR rounds.

Representing ethics reflection: If the allied key actors and the facilitator team included highly respected persons/HCPs, participants believed that it had a positive effect on the views on and credibility of ethics and ECR rounds, as one participant said: “I think that one contributing thing that is good is when Aron [fictive name] is involved as a facilitator. He is a doctor…I think it’s a… magnet. He promotes it a bit in his group…I think it’s an issue of PR [public relations].”

Using self-experienced ethics reflection: Another way to promote ethics was by having HCPs experience for themselves by inviting them to participate in an ECR round. Participants found that when HCPs had participated in an ECR round they wanted to join again. Because each ECR round also constituted a promoting activity, it was important that it was a positive experience for all involved. Even though the facilitator team, their allied key actors and HCPs were enthusiastic and excited about the ECR rounds, it remained hard to find time in the busy and stressful clinical daily life.

### Scheduling Ethics Reflection

Scheduling ethics reflection includes planning and blocking time for ECR rounds. This strategy includes scheduling ECR rounds at any random time but usually at a permanent day/time to reach a continuity/routine. It can also involve using existing meeting times, that is, using a meeting structure that already exists.

Scheduling ethics reflection was viewed as instrumental for the activity to be carried out. “we will have fixed dates for the autumn. Then you sort of blocked that time… Because if you haven’t written anything in the schedule, it becomes…very difficult”. Some participants struggled to get HCPs to take the time for ethics and thought that the clinic was not well dimensioned for this type of activity. Thus, scheduling ethics reflection was not always easy in this context, as one participant said, “sometimes you can have so much to do that you don’t even have time to go to the restroom… and so we still tried to fight for and set a date”.

### Identifying Ethical Dilemmas

Identifying ethical dilemmas includes identifying the relevant patient cases involving ethical dilemmas which causes worry, uncertainty, and conflicts. Furthermore, in the ECR round about the specific patient case, the relevant ethical dilemma needs to be identified. This is important for positioning ethics reflection because it influences the experience of the ECR round. What is viewed as relevant for positioning ethics is not only the most difficult dilemmas or the dilemmas causing most conflicts, but also the frequent everyday ethical dilemmas.

The key actors and the group of facilitators observed and listened to what was going in the clinic to identify ethical dilemmas. One participant said: “There are cases that you have picked up during the day when you have been at the ward, or during the week, perhaps. That you sit in the staff room and hear how they are buzzing.” Furthermore, it was important that the group of facilitators and the key actors represented different professional groups as they could easier identify ethical dilemmas important for the various groups. As one participant said “…[he] is a doctor, so he can… get a sense of what is happening in the medical group, and are there questions there.” Also, as HCPs gained own experiences of participating in ECR rounds, they could become key actors and identify cases/ethical dilemmas in the clinic. Participating in an ECR round concerning one patient gave rise to ideas of ethical dilemmas concerning other patients.

Participants expressed a need for reflecting more often on the everyday ethical dilemmas and not only the dilemmas related to treatment, and life and death. Participants believed that HCPs would feel better if they were able to reflect upon ethical dilemmas that arose in everyday clinical practice and that all HCPs in the team could be involved in the decision, for example regarding physical restraint of children. Participants expressed that there probably were several cases and ethical dilemmas that were not paid attention to, because HCPs did not recognise that there was an ethical dilemma in the case. Sometimes, it was easy to identify an ethical dilemma in a case because it was very clear. However, sometimes it was very hard and frustrating to identify the ethical dilemma in the case as it was confused with for example medical, emotional, or organisational dilemmas.

## Discussion

In this study, facilitator trainees’ experiences of implementing ECR rounds in childhood cancer care were explored via focus group interviews and individual interviews. In a context where direct patient care was prioritized, participants were positioning ethics reflection to resolve their main concern of doing ethics. Strategies for positioning ethics included allying, promoting ethics reflection, scheduling ethics reflection, and identifying ethical dilemmas.

That direct patient care is prioritised over ethics reflection is not surprising. This is in accordance with a previous review about missed care, which found that acute care needs of patients were prioritized (Papathanasiou et al., 2024). Furthermore, another review showed that medical treatment was prioritized, and nurses felt lonely in their effort to provide ethical care (Suhonen et al., 2018). Although direct patient care needs to be prioritized, we would argue that ethics reflection should be an obvious and integrated part of standard patient care, to secure an ethically good care. Thus, it is important to question the polarization between care and ethics. The polarization is more serious than just being busy and having no time for ethics reflection. As long as employees and managers see ECR rounds as luxury, and not as a natural part of patient care, it will remain difficult to position ethics. Although the facilitator trainees were members of the teams of HCPs in paediatric oncology, they were struggling for the ECR rounds to be integrated as part of standard care in clinical practice. Also in the Netherlands, integrative CES refers to the integration of CES in clinical practice, and they identified fundamental features of this approach (Hartman et al., 2020). In contrast, integrated ethics in studies from the United States aimed at the integration of different ethics support strategies, with a broad scope, and within a common structure (Danis et al., 2021; Fox et al., 2010). However, underutilization of CES services was found to be one of the greatest challenges, suggesting that it was not integrated as part of standard treatment/care (Danis et al., 2021).

The facilitator training of HCPs in paediatric oncology included practice in their clinical setting. This meant that they also needed to implement the ECR rounds, which is somewhat problematic because of the great workload. It is clear that participants in our study found it challenging to implement ECR rounds in their clinical settings, and that they used several strategies to positioning ethics. Positioning ethics in clinical practice has also been found to be a fundamental feature of integrated CES (Hartman et al., 2020). It can be questioned who is responsible for implementing ethics support in clinical practice; if it should be the trainees/HCPs, trainers/researchers, ethics support staff or the hospital management. Most likely it will depend on the context. A way to prepare the organization is to develop a concrete plan how to implement ECR rounds and who is responsible for what before the training. In that way it becomes a shared responsibility and will not be on the facilitator trainees’ shoulders only. The management team is important in promoting an ethical climate in the organization (Ventovaara et al., 2021). Thus, should allocate resources for ECR rounds as an integrated part of patient care and the clinical organization. This could be done, for example, by being involved in the development of the concrete plan for implementation, ensuring that employees have relevant competence, allocating time for ethics reflection in the healthcare team and promoting ethics reflections in their communication with employees. In Norway there are legal requirements for clinical ethics committees at all hospitals and then the management is responsible (Magelssen et al., 2020), but no such law exists in Sweden.

The results demonstrated that facilitator trainees used the strategy of “allying”. Since working together in interdisciplinary teams is a core competence in healthcare (Institute of Medicine, 2003), it could be seen as self-evident in healthcare. Allying could be compared the “Shared ownership” which was identified as an implementation strategy in a study conducted in the Netherlands (Weidema et al., 2016). Furthermore, “creating co-ownership” is part of an integrative approach to CES (Hartman et al., 2020). It seems that managers interest in ECR rounds was crucial for successful implementation. In a study performed in the Netherlands, results show that managers highly valued the use of ECR rounds among nurses as it encouraged their empowerment and stimulated them to engage in critical reflection, which the managers considered essential professional skills (Weidema et al., 2015). However, they found implementing ECR rounds to be difficult due to the confidential and equal nature of the process, leading to various ethical dilemmas. Despite the challenges, managers acknowledged the positive outcomes of ECR rounds in terms of the process, but they struggled to control or regulate these outcomes effectively (Weidema et al., 2015). Future research should continue to investigate ECR rounds’ impact on patient care and thereby be able to provide scientific evidence on specific outcomes.

The possibility of being able to identify ethical dilemmas was important for promoting ethics reflections because it affected the experience of the ECR round. After a positive experience the HCPs are likely to spread the word and thus become key stakeholders/advocates of the implementation process, which is similar to an important strategy in implementation research (Kitson et al., 1998). However, sometimes it was difficult to identify the ethical dilemma which caused frustration, and varying levels of ethical skills in the team has previously been identified as a barrier to ECR rounds (Bartholdson et al., 2018). We would argue that the frustration is a barrier to the implementation of ECR rounds. The results also showed that HCPs gained competence in identifying ethical dilemmas by participating. Increased ethical competence have been found to be an important goal of ECR rounds (Dauwerse et al., 2013) and an outcome that HCPs consider important (Weiner et al., 2021).

### Strengths and Limitations

The aim of classic grounded theory is to generate a middle-range theory, originally from large sociological studies (Glaser & Strauss, 1965, 1967). However, “Grounded theory can be presented as a set of propositions… using conceptual categories and their properties” (Glaser & Strauss, 1967, p. 31). The conceptualisation of the results in the current manuscript is kept close to the context, for example using “Positioning ethics” instead of just “Positioning”. However, “positioning ethics” could be relevant also in other contexts and not only in the healthcare context, however, modification might be needed after further theoretical sampling (Glaser, 1978). Furthermore, the present study included no observations which could be viewed as a limitation. However, interviews are commonly used in classic grounded theory studies and the method is independent of what kind of data used as “all is data” (Glaser & Strauss, 1967).

Deviations from the method of classic grounded theory could be seen as limitations of this study. Recording is not recommended in classic grounded theory because it undermine delimitation of data (Glaser, 1998). However, we decided to sound-record the interviews in order to have access to quotes from the participants and because several people were involved in the data collection. Furthermore, a software is not recommended for the analysis of data, but was used in this study for coding, however, the sorting of memos and theoretical coding was done manually using paper (Glaser, 1998).

## Conclusions

The study highlights the need of positioning ethics in care, enabling HCPs in doing ethics, and also explains strategies for doing so. It is not surprising that direct patient care is prioritised, however, it is important to question the polarisation between care and ethics and instead use strategies for positioning ethics within care and making it an obvious and integrated part of standard care.

To implement ECR rounds in care, strategies need to be used. It is evident that it is important not to be alone and to form an alliance of key actors in the implementation process. Furthermore, considering the challenging situation in healthcare with shortage of staff and resources, setting aside time (scheduling) for ECR rounds becomes absolutely crucial. Ethical competence seems to be central in doing ethics and is a possible effect among HCPs participating in ECR rounds. Future studies could continue to explore how ethical competence is best promoted in clinical practice and in education.

## Data Availability

Data will be made available upon reasonable request.
